# The War and the Poor-Law Infirmaries

**Published:** 1914-08-15

**Authors:** 


### THE WAR AND THE POOR-LAW INFIRMARIES.

The provision of accommodation for the treatment of our sick and wounded soldiers and sailors in the war is a matter which requires to be organised at once. As to the number of beds that are likely to be needed there are at present no data upon which even an approximate estimate can be formed. No war of the magnitude and under the conditions of the present one has ever been waged, at any rate since Napoleon's last campaign. All one can safely say is that the demand for beds will probably be enormous. Conflicting as the reports of the preliminary fighting have been, they all agree that the carnage has been very heavy. To the number of the wounded will soon have to be added those stricken down by the sickness which always breaks out when large armies are in the field. When our Expeditionary Force is in action on the Continent and when our Fleets are engaged we must be prepared to meet a great demand for hospital accommodation. Obviously, our sick and wounded on the Continent will have to be treated in the first instance in hospitals near the fighting-line, but after the first few weeks of the war all the disabled who can bear the journey must be returned to England in order to keep those hospitals ready to meet the influx of fresh acute cases. To these must be added the thousands of wounded and sick landed at our ports after any large naval action.

It is our hospitals in the towns on our East and South-East Coasts that will have to make the first provision. But to keep them clear for fresh patients arrangements must be made to draft those able to travel to hospitals inland. The voluntary hospitals have already placed hundreds of beds at the disposal of the Government, but they can only do so by excluding cases from the general population which they would ordinarily receive. Even so it is probable that the accommodation so far promised will prove inadequate to the demand. The problem to be faced in the near future is, therefore, the provision of a large number of additional beds for the Army and Navy and of beds for the civilian population displaced from the hospitals. It is in the solution of this problem that the Poor-Law infirmaries may have to give help.

A step in this direction has already been made by the Kingston-upon-Hull Guardians, who have offered their new infirmary to the Government. Other Boards of Guardians should at once follow this example, take stock of their existing accommodation, and state what beds they have at their disposal to meet the demands which must be met. Fortunately the number of beds which might be set free in our Poor-Law infirmaries for this purpose is very large. At this time of the year all the infirmaries have a large number of beds actually empty, and a still larger number occupied by chronic cases which, in an emergency, could be sent to their homes and treated by the district medical officers and by district nurses. The test of destitution which has hitherto governed the admission of patients to the Poor-Law infirmaries in all but a minority of the cases must obviously go by the board. The only test for admission must be the medical requirements of the case. There would be no practical difficulty on this ground, for the medical officers have the power to admit any urgent case without any formalities, and maintenance in a Poor-Law infirmary does not entail any disqualification. The only difficulty which would arise, apart from the waning prejudice against the Poor-Law infirmary resulting from errors of administration in the past, would be the insufficiency of the staff. Poor-Law infirmaries are staffed on the assumption that a large number of their inmates belong to the chronic and incurable class. Even under existing conditions the staffing is in most cases insufficient, and it would be impossible, without a large increase in the staff, to cope with the influx of a number of acute cases. This difficulty is, however, not an insuperable one, and in a time such as this the existing staff may be relied upon to reduce it to a minimum by submitting cheerfully to any necessary curtailment of off-duty time and leave of absence. In our Poor-Law infirmaries we have a well-equipped and well-organised system of hospitals ready and eager to meet any demands the Government may find it necessary to make upon them.

